# Exploring staff and service user experiences of personality disorder services in open prisons: A qualitative study of Pathways Enhanced Resettlement Support

**DOI:** 10.1371/journal.pone.0350292

**Published:** 2026-06-10

**Authors:** Georgina Mathlin, Hannah Jones, Carine Lewis, Claudia Cooper, Mark Freestone

**Affiliations:** 1 Centre for Psychiatry and Mental Health, Wolfson Institute of Population Health, Queen Mary University of London‌‌, London, United Kingdom; 2 His Majesty’s Prison and Probation Service, London‌‌, United Kingdom; 3 Tavistock and Portman NHS Trust, London, United Kingdom; Newcastle University, UNITED KINGDOM OF GREAT BRITAIN AND NORTHERN IRELAND

## Abstract

Re-entering the community following a prison sentence is a critical period for rehabilitation, especially for people in prison with a likely diagnosis of personality disorder. A joint health and criminal justice initiative across England and Wales, called the Offender Personality Disorder (OPD) Pathway, aims to support individuals with a likely diagnosis of personality disorder with services across different prison and community settings. Pathways Enhanced Resettlement Services (PERS) are OPD services operating in five open prisons in England, which aim to support people at high risk of being returned to closed conditions or reoffending in the community after release. We aimed to understand how service users and staff experience PERS. We purposively selected staff and service users within male prisons to encompass a range of staff roles and current and ex-service users. We conducted semi-structured interviews with ten staff and nine service users (seven current and two ex-service users). We conducted a reflexive thematic analysis, generating three themes: (1) “A shock to the system”: this theme describes the challenges of adapting to open prison culture as a liminal space between closed conditions and the community; and understanding and accepting PERS as a service to provide support within and beyond that space. (2) “We’ve got some understanding of their journey”: This theme describes how service users felt valued by PERS support that was centred around their needs, a personalised approach that enabled the development of trusting, therapeutic relationships. (3) “Internal states can be real barriers to progression”: this theme describes how PERS staff were able to use the therapeutic relationships they developed with service users to support them to progress through their sentence. Staff and service users felt that PERS provided support to progress through open prison, largely by developing positive trusting relationships and individualised support plans.

## Introduction

Re-entering the community from prison is a critical period for rehabilitating people who have been in prison. It is associated with social, economic and psychological difficulties, and increased risks of homelessness, unemployment, mental health difficulties and reoffending [1]. Support in the period before release from prison may ease the transition and reduce the risks to society and individuals.

Structured re-entry interventions delivered in the United States aim to support the transition into the community and reduce recidivism [2]. Some interventions focus solely on issues such as reducing substance misuse [3], whereas others target multiple areas such as housing, employment and social support [4]. A meta-analysis of nine randomised controlled trials exploring interventions for work and employment or multimodal interventions including support with housing, employment, mentoring and drug treatment, found a lack of consistent evidence that re-entry interventions reduce recidivism for adult males [1]. Some studies have reported on reintegration outcomes outside of recidivism, reflecting a more holistic view of progression from prison into the community, finding mixed evidence for the impact of re-entry interventions on housing, employment and social support [1]. Furthermore, there is limited research exploring the experiences of those taking part in re-entry interventions [5].

In England, the Offender Management in Custody sentence management approach plans the resettlement of a prisoner from the first day of custody through the shared responsibility across government agencies including the prison and probation service, local authorities and NHS England [6]. This system has come under scrutiny, with a recent government report indicating resettlement services are inadequate, leading to inequalities for those returning to the community due to ineffectiveness and inconsistencies in the support received [7]. Open prisons play an important role in this cross-agency resettlement approach. They house “Category D” adult males, who have a low risk of absconding and constitute a low risk to the public due to the nature of their offence, and those nearing the end of longer sentences transitioning from higher security categories, providing opportunities for Release on Temporary License (ROTL). ROTL allow periods of short-term release to the community, to facilitate successful community integration on release [8].

Relative to closed conditions, open prisons have a different culture: residents are afforded greater freedom and autonomy in exchange for greater behavioural expectations. This can be difficult for those who have become institutionalised in closed condition prisons [9–11]. Retaining a position in an open prison can be difficult, due to the low threshold for being returned to a closed condition establishment, including substance misuse lapses, feeling threatened or bullied and being violent [12]. The ease of being returned to closed conditions and witnessing people being returned can bring a sense of insecurity [13]. Danks and Bradley [14] found residents in open prisons were reluctant to seek support for mental health issues for this reason; and residents in open prisons have been reported to be reticent to use complaint systems due to fears of being perceived as problematic and removed [15].

### The resettlement of people in prison with personality disorder‌‌

An estimated two-thirds of the prison population in western countries meet personality disorder diagnosis criteria [16]. Personality disorder is a complex mental health condition, characterised by poor relationship skills, issues with identity and, in some cases, a risk of physical harm to others [17] which people likely experience varying severities of difficulties [18]. Within western prison populations, 47% of males have an antisocial personality disorder diagnosis [16], a type of personality disorder characterised by and increased risk of violence and recidivism [19] substance misuse and self-harm [20,21]. Working with individuals with personality disorder has been associated with high levels of burnout due to emotional and relational demands of this work on staff [22].

The Offender Personality Disorder (OPD) Pathway is a joint health and criminal justice initiative commissioned by His Majesty’s Prison and Probation Service and NHS England [23]. The pathway was set up in 2012 to support individuals with a high risk of recidivism, assessed as meeting criteria for personality disorder. It aims to reduce repeat serious offending, improve psychological wellbeing, produce a competent and trained workforce, and use resources efficiently. Evidence for the effectiveness of the OPD pathway is mixed with a national evaluation indicating self-perceived reductions of risk and increased wellbeing but inconclusive quantitative findings in relation to engagement with the pathway and reduced recidivism [24]. People are screened into the OPD pathway using an algorithm [25], designed to assess presence of personality disorder traits and risk, and subsequently receive a formulation to inform sentence planning and suggest engagement with OPD services if appropriate.

Open prisons are a particularly challenging environment for individuals with a likely diagnosis of personality disorder and therefore open prison OPD services known as Pathways Enhanced Resettlement Services (PERS) have been operational since 2019, focusing on individuals with a high risk of “failing” in the short-term, either through reoffending following release or being returned to closed conditions. PERS aim to support people in open prisons, reducing their risk of being returned to closed conditions and preparing for transition into the community, acknowledging that progression for this population may be non-linear. Service users join voluntarily and alongside usual open prison programmes, receive key work sessions with PERS prison officers where they develop a working formulation to anticipate difficulties and agree a management plan. The services have an open-door policy for individuals to ‘drop-in’ and receive ad hoc support and pre-empt crises.

Only one previous study has explored the experiences of PERS service users [26]; it focussed on the service when it was set up as a pilot in one open prison. The study highlighted the importance of developing trusting, supportive relationships with staff as a key mechanism for change. This reflects findings from other studies exploring OPD service user experiences [24,27]. PERS has since been implemented across five open prison sites. We conducted the first qualitative study to interview service users and staff from PERS in open prisons, and the first qualitative study to purposively recruit service users across the national networks of open prisons in England that include a PERS service. We aimed to explore how staff and service users experience PERS and how the service has, or has not, supported individuals to remain in open prison and transition successfully to the community.

## Materials and methods

### Study design

This study utilised a semi-structured qualitative‌‌ design underpinned by a critical realist methodological orientation [28]. We set out to understand what has, or has not, supported individuals to remain in open prison and transition successfully to the community (the mechanisms of PERS) as well as the context in which those mechanisms occur (through capturing multi-perspectives of staff and service users).

### Ethical approval

Ethical approval was granted by HMPPS National Research Committee (Ref: 2020−131/ Ref: 2021−180). All participants provided written informed consent.

### Procedure and sample

To explore the experiences of staff and service users we conduced semi-structured interviews. We recruited PERS staff from the five operational services, located in 5/11 adult male Category D (open) prisons in England. Between 1^st^ April 2021 and 30^th^ July 2021, we recruited ten of twenty staff (two staff members from each service were approached via email to take part in the study), purposively, for diversity of staff roles, levels of experience and clinical and operational staff. All staff that were approached to take part in the study agreed to participate and provided informed consent by completing the consent form and emailing it to the lead author before the interview began. Three groups of PERS service users were eligible to participate: ex-PERS service users in the community, current PERS service users and ex-PERS service users in closed conditions prisons. Potential participants were initially approached by PERS staff, directly or via offender managers of those returned to closed conditions between 7^th^ May 2022 and 1^st^ September 2022. Staff then arranged interviews for current service users and passed details of ex-service users living in the community willing to participate to the researcher. It was not possible to approach anyone in closed conditions prisons during the study period due to COVID restrictions and therefore none took part in the study. All participants who were approached by PERS staff took part in the study. Informed consent was obtained in person prior to the interview for current service users who were interviewed in person allowing opportunity to clarify anything before beginning. Individuals in the community completed the consent form and returned it to the lead researcher via email before the interview commenced and at the start of the interview this was verbally re-confirmed. Neither staff nor service users received any compensation for their participation.

To determine an appropriate sample size, the information power framework was utilised [29]. The narrow study aim, dense sample specificity and expected high quality of dialogue within the interviews indicated a smaller sample would be adequate. However, the lack of established theory of PERS and plan to analyse across cases suggested a larger sample would be most appropriate. Taking these criteria into consideration, a sample of 20 (ten staff and ten service users) was considered sufficient to address the research aim. The final sample size obtained was 19 (ten staff and nine service users).

### Data collection and processing

Interviews with PERS staff were conducted by the lead author (GM) remotely via Microsoft Teams between April and July 2021 whilst in-person research within prisons was prohibited due to COVID-19 restrictions. The co-authors developed a topic guide (S1), which explored respondents’ job role, and their views on how PERS supports users in open conditions; the interviewer probed potential barriers and facilitators of service users benefitting from PERS support. The topic guide also included questions regarding how COVID-19 impacted service delivery and service user outcomes. Interviews with PERS staff lasted on average 50 minutes.

Interviews with service users were conducted by the lead author in June and July 2022, with current service users interviewed in person at the open prisons and ex-service users interviewed remotely via telephone. We were unable to recruit any ex-service users in closed conditions. The topic guide (S2) developed for service user interviews asked how they had joined PERS, how PERS had been helpful or unhelpful, how COVID had impacted their use of PERS, what they felt they needed from PERS to succeed, their wellbeing whilst working with PERS and, if applicable, their experience of transitioning into the community from PERS. Interviews with service users lasted on average 45 minutes.

All interviews were recorded on an encrypted Dictaphone, transcribed verbatim by the lead author and no one else was present during any interviews aside from the lead researcher and the participant. No repeat interviews were carried out. Field notes were made immediately after each interview and reviewed as the initial stage of the analysis.

### Data analysis

We conducted a Reflexive Thematic Analysis (RTA) [30] using NVivo 14 [31] to code interview transcripts. RTA refers to the processes of critical reflection and refinement pursued by the researcher, during which subjectivity and nuance are considered in relation to predominant assumptions and socio-cultural context [32,33]. Reflexivity in this study manifested as iterative examination, adaptation and refinement of themes, initially generated by GM. Braun and Clarke’s [34] six phase process for RTA was used to analyse the interview data, which were coded inductively.

Phase 1 was *familiarisation* with the dataset. This involved re-listening to the recordings, becoming immersed in the data, and making notes with initial ideas. Phase 2 was *coding*; working through the dataset and applying labels to segments of text that appeared potentially relevant or meaningful. Phase 3 was *generating initial themes*, clustering codes into broader patterns and ideas. Phase 4 was *developing and reviewing themes*, whereby themes are re-assessed and compared with other themes and against the entire dataset. Phase 5 was *refining, defining and naming themes*. The final phase, 6, was the *writing up*.

GM carried out phase 1–3 of the RTA. The initial themes were developed, reviewed and refined alongside MF and HJ. Preliminary findings were shared via email with all PERS staff for comments and presented at a staff away day for discussion which aided the interpretation of the data. This supported us to develop to consider emerging themes. It was not possible to re-contact service users to check the themes. We included quotations – selected for their illustrative efficacy – to emphasise key points. Whilst all interviews contributed directly or contextually to our resulting themes, not all were quoted.

### Researcher reflexivity

GM, MF and HJ have previously worked as staff in OPD pathway services (although not in PERS) and had prior knowledge and expectations about the experiences of staff and service users which likely influenced the interview questions and the interpretation of data. The interviewer was a female PhD candidate, with some experience conducting qualitative research and of working in OPD services as an assistant psychologist which brought an understanding the OPD context that helped build rapport. Despite the interviewer not being a clinician within the prison, some service user participants may still have been inhibited in their discussions due to the interviewer holding the position of a researcher.

## Results

### Sample characteristics

No demographic data was collected about gender (for the staff, all service users were residents in male prisons), age or ethnicity of participants in line with the ethics agreement to maintain confidentiality in this sensitive area. Ten staff were interviewed (two from each service). We interviewed five clinical staff (clinical leads and an assistant psychologist), and five operational staff (mental health nurses and prison officers). The mental health nurses were considered part of the operational staff, due to their role within PERS aligning with the operational delivery of the service, such as providing key work sessions. Staff had worked in PERS between three months and two years (the clinical staff average was 13 months, and the operational staff average was 14 months). Three of the clinical staff and three of the operational staff had prior experience of working in other OPD Pathway services.

Nine service users were interviewed (two each from three of the services, one from another and two in the community at the time of interview). Seven were current PERS users and two were ex-PERS users living in the community. The service users had been in open prison between five months and three years. All service users had been in higher category prisons prior to entering open prison, two of the current service users had been in open prison previously (and been returned to closed conditions) and two service users had been recalled from the community during their sentence.

### Qualitative findings

We developed three themes that responded to our research aims. Due to the constraints of our ethics permissions, we do not provide further demographic details of service users (denoted as SU after quotes); staff are described as operational (non-clinical staff with professional roles aligned with the prison service, denoted as OS) or clinical (clinical staff with training and professional roles aligned with health care: denoted as CS).

#### Theme one: “A shock to the system”: Understanding and accepting support from PERS within the liminal space of open prison.

This theme describes the challenges of adapting to open prison culture as a liminal space between closed conditions and the community of open prison; and understanding and accepting PERS as a service to provide support within and beyond that space. Service users described adaptation to open prison culture as “*hard work” [SU4]*, “*difficult” [SU7]* and “*strange” [SU5].* They struggled to navigate the processes for obtaining support: “*not knowing where to seek help, how to ask for help here” [SU5].* The different routine of open prison compared to closed conditions, especially the increased freedom, was described by a service user recently transferred from closed conditions as “*a shock to the system” [SU2]*. Alongside adapting to the freedom of open prison, service users expressed the precarity of this freedom. One participant commented: “*you say the wrong thing to a member of staff, you could get a nicking and then you’re going to get a ROTL ban” [SU4].* PERS staff also stressed how challenging the culture of open prison, with its greater autonomy and need for self-control was for service users arriving from closed conditions. For example, “*it’s really easy for them to get drugs if they want” [OS1]* and there’s “*less staff, less security, less everything to stop that” [OS1].*

The initial motivation for some service users to join PERS was because they were “*searching for that person to talk to, to understand how I was feeling… I wasn’t getting that via the units” [SU5].* Service users felt having “*somebody to talk to about how I was adapting” [SU9]* and being able to discuss concerns with PERS staff was helpful: “*coming here and letting that off to them, I find it real helpful” [SU5].*

Other service users expressed scepticism about PERS, with one participant commenting “*it’s a set up this” [SU4]*. Another participant commented “*I was a bit sceptical, thinking hold on a minute, does this mean I’m going back on the bus and I’m going back to closed conditions” [SU6].*

Staff reflected on the initial wariness of some service users approached to join PERS, stating the importance of encouraging voluntary engagement “*the service is voluntary; it therefore is quite non-threatening which enables people to engage with us” [CS4].* When service users were given more information about PERS and told that they had a choice to engage, they felt more open to joining “*the more I was chatting with them, I was thinking yeah you know what, they’re actually not bad” [SU4].*

#### Theme two: “We’ve got some understanding of their journey” increasing trust and meaningful engagement with service users.

This theme describes how service users appreciated and felt valued by PERS support that was centred around their needs, which contrasted with the more institutionalised, less personalised approach outside the PERS service. Providing emotional containment facilitated the development of trusting, therapeutic relationships.

Service users were positive about being able to use drop-ins “*you can just knock the door and walk in, and anyone of them will be happy to give you some advice and encourage you” [SU2].* Operational staff described providing personalised practical support: “*a lot of the guys don’t know how to use a mobile phone, we take that individual on a day out, we’ll go through it…, there’s no panic” [OS1].* Group activities such as tea and coffee evenings “*made me feel normal” [SU5]*. There was a clear emphasis that staff have time to provide this support to service users “*even if they’ve got a busy day, they’ll take the time out” [SU4].*

Service users reported having positive, trusting relationships with PERS staff where they felt “*relaxed around them, more open, honest” [SU6].* PERS staff were described as “*friendly and helpful” [SU1]* who “*listen to what you have to say” [SU8].* Service users described how they used PERS to offload worries “*I come up, I vent, I vent, I vent, and then I leave here and it’s just like, done’ [SU5].*

Service users described PERS staff as being available “*I can come over here any time of day” [SU4]* which was different to how they experienced non-PERS staff in open prison, who were described as “*not very helpful” [SU7].* For example, one participant described how they often feel dismissed when approaching standard wing officers to have a chat “*[they say] ‘you’ll be alright mate, don’t worry about it’ and close you behind your door when in reality, you just need a chat for ten minutes” [SU6].* Service users felt misunderstood by non-PERS staff “[*the wing officers were*] *looking at me like I’m speaking a foreign language” [SU7].*

Staff felt building relationships with service users was important for progression, whilst reflecting on how difficult it can be for the service users to develop trusting relationships “*a lot of them don’t trust” [OS6]*. They spoke about providing positivity “*giving people praise” [OS6]* and space to offload their worries as elements to relationship building otherwise lacking in service users lives. A clinical staff member commented “*it’s that emotional containment that feels important*” *[CS2]*, which they felt was developed through consistent key work and having drop-ins where service users receive ad-hoc support.

PERS staff recognised that the relationships service users develop with PERS staff were more trusting than non-PERS staff: “*We develop a far better…relationship with them than…the wing staff, sadly” [OS3]*. PERS staff feel they are “*more geared with emotional support” [OS4]* and have “*more time to help” [OS4].* Staff also felt they had a better understanding of service users than the general prison staff “*we’ve engaged with them in a different way, we’ve got some understanding of their journey…that in general the prison staff don’t have” [CS2].*

#### Theme three: “Internal states can be real barriers to progression”: Understanding and overcoming barriers.

This theme describes how PERS staff were able to use the therapeutic relationships they developed with service users to support them to progress through their sentence, especially around key events such as upcoming parole boards. Service users acknowledged how PERS “*try to move people forward through the system” [SU1].* One participant reflected how their contact with PERS increased as they approached their parole board, to get support with their anxiety “*I see them every week now, because of the anxiety” [SU5].*

PERS staff felt that the “*internal states can be real barriers to progression” [CS1]* such as “*anxiety”* and when individuals have become “*hopeless and disempowered” [CS4]* which may have developed due to having a limited sense of control. Service users reflected on how they felt they were able to overcome some of the internal barriers to progression by working with PERS. For example, one participant described how he “*used to class myself as scum” [SU5]* and felt no matter what was said, he had always previously felt like this. Some other service users described how “*most of my goals and my targets haven’t really been realistic or achievable” [SU7]* and that they use to be “*angry and I’d take my frustration out on staff or another prisoner” [SU5]*. However, through working with PERS, service users felt they had been able to move past some of these difficult internal states. For example, “*one of the biggest things for me, was never forgiving myself” [SU5].* Service users felt they were “*a lot more aware of how my actions affect others”* and described being able to “*set realistic targets” [SU7]* and as a consequence service users experienced being “*a lot more happier” [SU5]* following their engagement with PERS. This need to set expectations around progressions was also highlighted by staff: “*being really, really realistic with people… helping them structure expectations” [CS4]* was important when service users first enter open prison and that managing service user expectations of open prison helped them adapt*.*

Staff acknowledge however that external as well as internal conditions influenced progression. Notably, progression during COVID-19 was impacted as “*people were not getting parole because they hadn’t been able to access ROTL, essentially what they’ve come to open conditions to do” [CS4]*. However, some staff did also reflect that “*[service users] got released when they’d had just had a couple of ROTLs, which never user to happen [before COVID]” [OS6].* Although it was positive to see that some people were able to progress in this time, there was also concern that individuals released without utilising ROTL may have missed out on opportunities to “*gain qualifications”* and “*bolster their support networks” [OS5]*.

### Reflective statement

Staff and service users were willing and open to participate in the study and explore their experiences of PERS and how they felt PERS influences progression. Staff generally had similar views about their experiences of implementing PERS and the factors that influence progression, however, clinical staff tended to focus on the wider systems that service users exist within and the emotional difficulties they experience, whilst operational staff provided more detail on the day-to-day service delivery and the practical support they provide. This is likely to reflect their roles and the type of contact they have with the service users. Some service users expressed their dislike toward authority and directly referenced the interviewer being an authority figure that previously they would not have felt comfortable speaking with. They stated the work they had done with PERS allowed them to be more open with authority figures and therefore take part in these interviews.

## Discussion

This is the first study to explore experiences of staff and service users across all five PERS services. The themes we generated described the challenges service users experienced in adapting to open prison culture as a liminal space between closed conditions and the community; and understanding and accepting PERS as a service to provide support within and beyond that space. The themes demonstrate initial mistrust towards PERS, mitigated by development of trust through support centred around their needs, with this trust then influencing engagement in later stages whereby PERS staff were able to use the therapeutic relationships they developed with service users to support them to progress through their sentence.

### Difficult transitions into open prison

Many of the service users’ motivation to engage with PERS was to gain support with the transition into open prison, but some service users initially felt suspicious about what PERS was. The reluctance to engage with PERS supports research exploring factors which may impact the readiness to engage in services [35]. The suspicion and reluctance to engage in PERS highlights potential negative perceptions of the service, which is likely linked to a lack of trust in others [35] and in systems more widely. PERS staff reduced barriers to engaging by providing clear information about the service and a providing a choice about whether to engage. This in turn allowed service users to start to develop trust with PERS staff.

Moving to open prison was described as a difficult transition by all service users interviewed, with many struggled to adapt to the increased amount of freedom. Previous research has reported service users feeling overwhelmed when moving to an open prison [13]. PERS staff were aware of how difficult this transition could be and reported managing expectations as a useful tool to help service users adapt to their new environment. Expectations of treatment have been explored as a factor linked to treatment non-completion in individuals with personality disorder [35] but have not been considered in relation to standard prison transitions.

### Relationships with staff

Service users and staff highlighted the positive trusting relationships they experienced working with PERS. Building trusting relationships has been a consistent theme related to change in other OPD pathway services [36,37] and a pilot PERS service [26]. Trusting relationships were seen as a key mechanism for progression within PERS.

Many staff and service users spoke about poor relationships with non-PERS staff in the open prisons, such as feeling misunderstood by non-PERS wing officers. Poor staff-service users relationships have been reported in previous research in open prisons [10,13,14], linked to the precarity of privileges and the potential for staff to remove them. Poor relationships with staff in open prisons are especially concerning for those with a likely diagnosis of personality disorder, who often experience difficulties with interpersonal functioning and authority [38]. A broad aim of the OPD pathway is workforce development [39] with the added benefit of systemic change in the wider environments that OPD services operate within. PERS staff should therefore focus further on providing supervision and support to prison staff in open prisons to help develop better relationships with service users.

### Progression through open prison

PERS was felt to be supporting progression through the open prison and preparing people for release into the community. Several barriers to progression were noted by staff and service users. The accessibility of drugs in the open prison estate and the reduced levels of security to monitor this were noted by staff. Low levels of hope and control were also considered as barriers to progression. There are associations between externalising control and offending behaviour [40], with greater internal control associated with hopefulness [41]. Having hope and a sense of internal control are likely to aid people through the difficulties of progressing in open prison and into the community [36]. Increasing levels of hope and control is therefore an important task of PERS and was seen through many service users reporting increased wellbeing, a newfound ability to forgive themselves and the ability to set more realistic goals. PERS is modelling the OPD pathway approach well, which intends to provide support across the various stages of a person’s journey through the Criminal Justice System [42]. This is especially important in relation to the context of open prison, an important stage of progression in many offender journeys that can be difficult to manage [10].

### Limitations

PERS staff may have been biased in reporting the positive impact of PERS, as they had an interest in the continuation of the service. Likewise, the sampling of service users through the PERS staff may have biased sampling towards those who felt positively towards the service. It was not possible to recruit ex-service users returned to closed conditions in the current study, and they may they had different, less positive experiences. It was also not possible to report demographic information, including ethnicity and therefore we were not able to comment on how experiences across may have differed across ethnic groups or the representativeness of the sample. This research took place in the pandemic, a unique time which provides critical insights into experiences at this time, though may also limit generalisability. The number of prison releases following the COVID-19 pandemic (March 2020) reduced by 15% compared to the same period in 2019 [43] but this may be attributable to the prison population serving longer sentences, rather than a direct effect of COVID.

### Conclusions

Despite this study exploring low security open prisons, it is promising that the provision of individualised support, addressing needs on a person-by-person basis was received positively by service users and staff; while our findings cannot necessarily be generalised beyond this setting, findings may inform approaches across the prison estate. Further consideration should be given to the relationships developed between prisoners and prison staff, when trusting relationships are developed individuals appear more willing to engage with interventions and receive support in a meaningful way.

## Supporting information

S1 Table Staff interview schedule. (DOCX)

S2 Table Service users interview schedule. (DOCX)
