# Supplementary material for: Exploring staff and service user experiences of personality disorder services in open prisons: A qualitative study of Pathways Enhanced Resettlement Support
Source: PLoS One. 2026 Jun 10;21(6):e0350292. doi: 10.1371/journal.pone.0350292 (PMC13252747; doi:10.1371/journal.pone.0350292)
Supplement: S1 Table — (DOCX) [file pone.0350292.s001.docx]

**Supplementary Materials 1:**

**Staff Interview Schedule:**

| Topics | Main Questions |  | Follow-up Questions | | | |
| --- | --- | --- | --- | --- | --- | --- |
| 1. Introduction of myself/ the topic/ boundaries of the interview/ consent. |  |  |  | | | |
| 1. What is your job role? | How long have you worked in PERS? Did you have experience of working with OPD population/service prior to being in the PERS? | Can you tell me about what your job within the PERS is? | | What do you do on a day-to-day basis? | How much contact do you have with PERS service users on a weekly basis? |  |
| 1. What factors do staff think are important in the success of PERS offenders? | What do you think contributes to PERS user’s success in open condition? | Do you think the environment of the PERS service user, or the individual characteristics of the of the person are more important for their success? | | How does PERS help service users stop being returned to closed conditions? |  |  |
| *Show the logic model & SR outcomes* |  |  | |  |  |  |
| 1. What do PERS staff think of an I-DAG of progression factors? | What do you think of these diagrams? | Do the diagrams represent your understanding of what may be involved with progression? | | Is there anything you would add to or change about the diagrams? |  |  |
|  |  |  | |  |  |  |
| 1. What do PERS staff think of the logic model? | What do you think of the PERS diagram? | Does the diagram represent your understanding of how PERS works? | | Is there anything in the PERS structure that you would add or change? | Is there anything in the PERS outcomes that you would add or change? | Are there any contextual factors you think are missing? |
| 1. How is PERS being impacted by COVID in both service delivery and outcomes for offenders? | How is the coronavirus outbreak impacting service delivery and service user outcomes? | What parts of PERS have you not been able to deliver due to COVID? | Do you think there will be long-term implications for service users due to coronavirus? | | | |
| 1. Thanks, and closing comments – opportunity to request notes/ transcripts. |  |  |  | | | |
