# Supplementary material for: Exploring staff and service user experiences of personality disorder services in open prisons: A qualitative study of Pathways Enhanced Resettlement Support
Source: PLoS One. 2026 Jun 10;21(6):e0350292. doi: 10.1371/journal.pone.0350292 (PMC13252747; doi:10.1371/journal.pone.0350292)
Supplement: S2 Table — (DOCX) [file pone.0350292.s002.docx]

**Supplement 2:**

**Service users interview schedule:**

| Questions |
| --- |
| Opening Question |
| How did you get here?* |
| Follow-up Questions |
| How has PERS been helpful? |
| How has PERS not been helpful? |
| How did COVID-19 impact your time at PERS? |
| What do you feel is needed for you to succeed? |
| Did you feel you were in a good place within yourself whilst at the PERS?  How is your mood now? |
| Community only: How was the transition from open conditions into the community? |

*Here will replaced for the situation the service user is in (closed conditions, open conditions, community)
