# Supplementary material for: Exploring staff and service user experiences of personality disorder services in open prisons: A qualitative study of Pathways Enhanced Resettlement Support
Source: PLoS One. 2026 Jun 10;21(6):e0350292. doi: 10.1371/journal.pone.0350292 (PMC13252747; doi:10.1371/journal.pone.0350292)
Supplement: Peer review history

# Peer review: Decision Letter 0

**DOI:** 10.1371/journal.pone.0350292.r001

**Authors:** Steph Scott

18 Mar 2026

PONE-D-25-20906

Exploring staff and service user experiences of personality disorder services in open prisons: A qualitative study of Pathways Enhanced Resettlement Support

PLOS One

Dear Dr. Mathlin,

Thank you for submitting your manuscript to PLOS ONE. After careful consideration, we feel that it has merit but does not fully meet PLOS ONE’s publication criteria as it currently stands. Therefore, we invite you to submit a revised version of the manuscript that addresses the points raised during the review process.

Detailed reviewer reports are included below. Please submit your revised manuscript by May 02 2026 11:59PM. If you will need more time than this to complete your revisions, please reply to this message or contact the journal office at plosone@plos.org. When you're ready to submit your revision, log on to https://www.editorialmanager.com/pone/ and select the 'Submissions Needing Revision' folder to locate your manuscript file.

Please include the following items when submitting your revised manuscript:

- A letter that responds to each point raised by the academic editor and reviewer(s). You should upload this letter as a separate file labeled 'Response to Reviewers'.
- A marked-up copy of your manuscript that highlights changes made to the original version. You should upload this as a separate file labeled 'Revised Manuscript with Track Changes'.
- An unmarked version of your revised paper without tracked changes. You should upload this as a separate file labeled 'Manuscript'.

If you would like to make changes to your financial disclosure, please include your updated statement in your cover letter. Guidelines for resubmitting your figure files are available below the reviewer comments at the end of this letter.

If applicable, we recommend that you deposit your laboratory protocols in protocols.io to enhance the reproducibility of your results. Protocols.io assigns your protocol its own identifier (DOI) so that it can be cited independently in the future. For instructions see: https://journals.plos.org/plosone/s/submission-guidelines#loc-laboratory-protocols. Additionally, PLOS ONE offers an option for publishing peer-reviewed Lab Protocol articles, which describe protocols hosted on protocols.io. Read more information on sharing protocols at https://plos.org/protocols?utm_medium=editorial-email&utm_source=authorletters&utm_campaign=protocols.

We look forward to receiving your revised manuscript.

Kind regards,

Steph Scott

Academic Editor

PLOS One

Journal requirements:

When submitting your revision, we need you to address these additional requirements.

1. Please ensure that your manuscript meets PLOS ONE's style requirements, including those for file naming. The PLOS ONE style templates can be found at

https://journals.plos.org/plosone/s/file?id=wjVg/PLOSOne_formatting_sample_main_body.pdf and

https://journals.plos.org/plosone/s/file?id=ba62/PLOSOne_formatting_sample_title_authors_affiliations.pdf

2. Thank you for stating the following in the Funding Section of your manuscript:

“This work was supported by the ESRC through Georgina Mathlin’s PhD funders, LISS-DTP.”

We note that you have provided funding information that is not currently declared in your Funding Statement. However, funding information should not appear in the Acknowledgments section or other areas of your manuscript. We will only publish funding information present in the Funding Statement section of the online submission form.

Please remove any funding-related text from the manuscript and let us know how you would like to update your Funding Statement. Currently, your Funding Statement reads as follows:

“GM

ESRC LISS-DTP PhD Studentship

https://liss-dtp.ac.uk/

The sponsers played no role in the study design, data collection and analysis, decision to publich, or preperation of the manuscript”

Please include your amended statements within your cover letter; we will change the online submission form on your behalf.

3. We note that you have indicated that there are restrictions to data sharing for this study. PLOS only allows data to be available upon request if there are legal or ethical restrictions on sharing data publicly. For more information on unacceptable data access restrictions, please see http://journals.plos.org/plosone/s/data-availability#loc-unacceptable-data-access-restrictions.

Before we proceed with your manuscript, please address the following prompts:

a) If there are ethical or legal restrictions on sharing a de-identified data set, please explain them in detail (e.g., data contain potentially identifying or sensitive patient information, data are owned by a third-party organization, etc.) and who has imposed them (e.g., a Research Ethics Committee or Institutional Review Board, etc.). Please also provide contact information for a data access committee, ethics committee, or other institutional body to which data requests may be sent.

b) If there are no restrictions, please upload the minimal anonymized data set necessary to replicate your study findings to a stable, public repository and provide us with the relevant URLs, DOIs, or accession numbers. For a list of recommended repositories, please see

https://journals.plos.org/plosone/s/recommended-repositories. You also have the option of uploading the data as Supporting Information files, but we would recommend depositing data directly to a data repository if possible.

We will update your Data Availability statement on your behalf to reflect the information you provide.

If the reviewer comments include a recommendation to cite specific previously published works, please review and evaluate these publications to determine whether they are relevant and should be cited. There is no requirement to cite these works unless the editor has indicated otherwise.

[Note: HTML markup is below. Please do not edit.]

Reviewers' comments:

Reviewer's Responses to Questions

**Comments to the Author**

1. Is the manuscript technically sound, and do the data support the conclusions?

The manuscript must describe a technically sound piece of scientific research with data that supports the conclusions. Experiments must have been conducted rigorously, with appropriate controls, replication, and sample sizes. The conclusions must be drawn appropriately based on the data presented.

Reviewer #1: Yes

Reviewer #2: Yes

**********

2. Has the statistical analysis been performed appropriately and rigorously?

Reviewer #1: N/A

Reviewer #2: N/A

**********

3. Have the authors made all data underlying the findings in their manuscript fully available?

The PLOS Data policy requires authors to make all data underlying the findings described in their manuscript fully available without restriction, with rare exception (please refer to the Data Availability Statement in the manuscript PDF file). The data should be provided as part of the manuscript or its supporting information, or deposited to a public repository. For example, in addition to summary statistics, the data points behind means, medians and variance measures should be available. If there are restrictions on publicly sharing data—e.g. participant privacy or use of data from a third party—those must be specified.

Reviewer #1: No

Reviewer #2: No

**********

4. Is the manuscript presented in an intelligible fashion and written in standard English?

PLOS ONE does not copyedit accepted manuscripts, so the language in submitted articles must be clear, correct, and unambiguous. Any typographical or grammatical errors should be corrected at revision, so please note any specific errors here.

Reviewer #1: Yes

Reviewer #2: Yes

**********

5. Review Comments to the Author

Please use the space provided to explain your answers to the questions above. You may also include additional comments for the author, including concerns about dual publication, research ethics, or publication ethics. (Please upload your review as an attachment if it exceeds 20,000 characters)

Reviewer #1: Overall, this was a well written, novel, and informative study on staff and service user experiences of OPD Pathway PERS. A clear rationale was evident for the current study, and sound justification for various research methods. The findings were well presented, and nicely contextualised. Below are some quite specific thoughts and minor recommendations.

Thanks, and best wishes!

1. Some typos – 36 (should ‘service’ be ‘service users’ here?); very occasional and minor grammatical errors (141, 355).

2. 52-53 – I’d be interested in knowing immediately from reading this how many papers/interventions were covered in this meta-analysis. Think important here to note albeit important but equally quite limited/blunt measurements of success. Have there been any qualitative studies? Worth mentioning here?

3. 59-61 – Does inequality here reflect the differential quality of resettlement services? Not quite sure of the point here.

4. 80-84 – I have no doubt you will have debated such an issue, but current thinking is moving towards conceptualising PD as more difficulties in self and interpersonal functioning (on a spectrum) rather than a fixed mental health condition. I would also provide some more explicit reasoning around why staff burnout is associated with working with PD, for example ‘due to the particular emotional and relational demands of this work...’.

5. 87-88 – This ‘with a likely diagnosis of pd’ is a bit vague.... perhaps ‘assessed as meeting criteria for a PD’.

6. A wider point about PD is that you are referring to PD very broadly; we know, of course, that certain PDs are more prominent in the CJS and in men (albeit potentially due to gendered diagnostic criteria). I do appreciate your aim for conciseness in this introduction, but I also wonder, given the variety of symptoms/presentations across various PDs, whether it might be worth just noting more particular prevalence and symptomatology of certain PDs in the male custodial setting.

7. 85-92 – Worth just noting briefly the state of the evidence base for OPD?

8. 90 – ‘Using an algorithm’.....‘designed to assess presence of PD traits and risk’?

9. 175-179 – If CL was involved in analysis, would her OPD experience not also be relevant here?

10. 186 – Is a mental health nurse really considered operational, rather than clinical, staff? Obviously, this would also change your results section, and how you denote participants.

11. The overarching theme title for theme two feels geared towards staff actions/experience/attitudes, but this theme also seems to encapsulate how service-users see and approach staff differently in PERS services. This may work as a kind of virtuous circle (whereby the more trusting staff appear, the more service users meaningfully engage, thereby increasing how trusting staff appear....). I just wonder if the theme heading could more explicitly acknowledge this change in behaviour/attitude from SUs as well as staff.

12. Just wonder, in the reflective statement, if there were any differences in the attitudes between service users still within PERS, and ex-service users, now in the community.

13. 335 – ‘In others’......‘and in systems more widely!’

14. 336 - Indeed. I felt the themes were, to some extent, presented in chronological order, with seemingly initial mistrust towards PERS mitigated by development of trust, with this then influencing engagement in later stages of the ‘rehabilitation’ and ‘release’ process (e.g., parole). Again, can’t help but consider the idea of a ‘virtuous cycle’ here. Just a reflection!

15. Limitations – While I appreciate why additional demographic data were not collected, it would be helpful to acknowledge the absence of ethnicity information, particularly for SUs. Due to this omission, obviously the paper cannot comment on known demographic skews within OPD service user populations (typically over-representing white individuals) or consider potential differences in experiences across ethnic groups.

16. 387-388 – Whilst I agree, I do think it would be important to caveat that this paper looked at quite a specific part of the prison estate.

Reviewer #2: Thank you for the opportunity to review this paper, and my congratulations to the authors on this novel work to explore the experiences of staff and service users of supports for people in open prisons with personality disorders. Below I have included some thoughts for your consideration, I hope they are helpful.

Overall

1. Acknowledging the exception for the name of programs (such as the OPD pathway), I strongly recommend the authors replace the word “offender(s)” throughout the paper and instead use a person-centred alternative, consistent with practices for reducing stigma, prejudice and harm in research and healthcare. (for example, in the abstract, first line of the introduction, page 4 line 78…) The papers below may be helpful resources.

Tran NT, Baggio S, Dawson A, et al. Words matter: a call for humanizing and respectful language to describe people who experience incarceration. BMC International Health and Human Rights. 2018.

Bedell PS, Spaulding AC. The Names Have Been Changed to Protect the . . . Humanity: Person-First Language in Correctional Health Epidemiology. American Journal of Epidemiology. 2018.

Introduction

2. P5L96 This sentence uses “high-risk” twice, it’s not clear to me what the first use to label people means. If the authors could please revise to clarify who the program is meant to serve (“high risk” individuals is also used on P4L87, if the authors could please clarify, high risk of/for what?).

Methods

3. More details on participants selection and the process of conducting interviews and informed consent are needed (Please see COREQ items 9-23). A few key examples of needed details:

- How were staff members selected and approached to participate? How many were invited and how many declined to participate?

- How were potential participants selected and invited to participate? How many people declined to participate? How was informed consent ensured (was the consent form reviewed verbally?). How was confidentiality protected during the interviews?

- Were people compensated for their participation?

4. (L154) The data analysis section is written in the plural first person, but there is no stage that describes any author besides GM being engaged in the work. Multiple coders are not required by RTA but suggest revising to be more explicit that there was no other input or review.

5. (L171) I think it would be helpful for authors to include why member checking was conducted only with staff and not program participants.

6. The section on researcher positionality requires more details to be consistent with COREQ guidelines:

- Specifically more details are required about what it means that co-authors “previously worked’ in OPD pathway services (as staff or care providers?)

- Key details about interviewer positionality and potential dynamics (such as gender, occupation, experience with qualitative research and relationship to correctional authorities) would also be important to include, particularly given that the authors report later that participants viewed the interviewer as an authority figure.

Results

7. The results section is missing description of how many programs were represented by current and former service users

8. Theme three (L273) is focused on the idea of progression which is a different framing from the binary (fail/succeed in open prison) used throughout the rest of the paper. I might suggest either revising the introduction to expand on the idea of progression or to contextualize the idea of progressing within the fail/succeed framework in the results section.

**********

6. PLOS authors have the option to publish the peer review history of their article (what does this mean?). If published, this will include your full peer review and any attached files.

If you choose “no”, your identity will remain anonymous but your review may still be made public.

**Do you want your identity to be public for this peer review?** For information about this choice, including consent withdrawal, please see our Privacy Policy.

Reviewer #1: **Yes:** Anya Frude

Reviewer #2: No

**********

[NOTE: If reviewer comments were submitted as an attachment file, they will be attached to this email and accessible via the submission site. Please log into your account, locate the manuscript record, and check for the action link "View Attachments". If this link does not appear, there are no attachment files.]

To ensure your figures meet our technical requirements, please review our figure guidelines: https://journals.plos.org/plosone/s/figures

You may also use PLOS’s free figure tool, NAAS, to help you prepare publication quality figures: https://journals.plos.org/plosone/s/figures#loc-tools-for-figure-preparation.

NAAS will assess whether your figures meet our technical requirements by comparing each figure against our figure specifications.

# Author comment: Author response to Decision Letter 1

**DOI:** 10.1371/journal.pone.0350292.r002

27 Apr 2026

Dear editor,

Thank you for considering our paper for publication. We have amended the manuscript to adhere with this style requirements, including aligning the funding information and data availability statements with journal requirements.

We respond to reviewer comments below.

Reviewer #1: Overall, this was a well written, novel, and informative study on staff and service user experiences of OPD Pathway PERS. A clear rationale was evident for the current study, and sound justification for various research methods. The findings were well presented, and nicely contextualised. Below are some quite specific thoughts and minor recommendations. Thanks, and best wishes!

Thank you for the time taken to review the manuscript.

1. Some typos – 36 (should ‘service’ be ‘service users’ here?); very occasional and minor grammatical errors (141, 355).

Thank you for identifying these typos. We have made these amendments: ‘service users on line 35, respondents’ on line 141 and ‘experience’ on line 355.

2. 52-53 – I’d be interested in knowing immediately from reading this how many papers/interventions were covered in this meta-analysis. Think important here to note albeit important but equally quite limited/blunt measurements of success. Have there been any qualitative studies? Worth mentioning here?

Thank you for this suggestion, we have added those details on line 52-55:

“A meta-analysis of nine randomised controlled trials found a lack of consistent evidence that re-entry interventions (encompassing support for employment, housing, and engagement with substance misuse services or mentoring) reduced recidivism among adult males (1)”.

We have also added on line 59-60: “Furthermore, there is limited research exploring the experiences of those taking part in re-entry interventions (5)”.

3. 59-61 – Does inequality here reflect the differential quality of resettlement services? Not quite sure of the point here.

We have expanded the sentence to clarify are argument on line 61-65:

“This system has come under scrutiny, with a recent government report indicating resettlement services are inadequate, with inequalities in the support received by those returning to the community (6)”

4. 80-84 – I have no doubt you will have debated such an issue, but current thinking is moving towards conceptualising PD as more difficulties in self and interpersonal functioning (on a spectrum) rather than a fixed mental health condition. I would also provide some more explicit reasoning around why staff burnout is associated with working with PD, for example ‘due to the particular emotional and relational demands of this work...’.

Thank you. We have added the following on line 83-86:

“Personality disorder is characterised by poor relationship skills, issues with identity and, in some cases, a risk of physical harm to others (16); it leads to difficulties in self and interpersonal functioning, which are often conceptualised as a being present as a spectrum within the general population, with those identified as experiencing personality disorder requiring treatment services in prison representing the most severe end of this spectrum (17)”.

We have added more explicit reasoning about staff burnout in relation to personality disorder on line 87-90:

“Working with individuals with personality disorder has been associated with high levels of burnout due to the emotional and relational demands of this work on staff (21).”

5. 87-88 – This ‘with a likely diagnosis of pd’ is a bit vague.... perhaps ‘assessed as meeting criteria for a PD’.

Thank you. We have changed line 99-100: “The pathway was set up in 2012 to support high risk individuals assessed as meeting criteria for personality disorder”.

6. A wider point about PD is that you are referring to PD very broadly; we know, of course, that certain PDs are more prominent in the CJS and in men (albeit potentially due to gendered diagnostic criteria). I do appreciate your aim for conciseness in this introduction, but I also wonder, given the variety of symptoms/presentations across various PDs, whether it might be worth just noting more particular prevalence and symptomatology of certain PDs in the male custodial setting.

We have added on line 86-88: “Within western prison populations, 47% of males have an antisocial personality disorder diagnosis (15), a type of personality disorder characterised by and increased risk of violence and recidivism (18) substance misuse and self-harm (19,20).”

7. 85-92 – Worth just noting briefly the state of the evidence base for OPD?

Thank you for this suggestion, we have added the following on lines 104-107:

“Evidence for the effectiveness of the OPD pathway is mixed with a national evaluation indicating self-perceived reductions of risk and increased wellbeing but inconclusive quantitative findings regarding engagement with the pathway and reduced recidivism (23).”

8. 90 – ‘Using an algorithm’.....‘designed to assess presence of PD traits and risk’?

Thank you. We have added to line 107-110:

“People are screened into the OPD pathway using an algorithm (24), designed to assess presence of personality disorder traits and risk, and subsequently receive a formulation to inform sentence planning and suggest engagement with OPD services if appropriate”.

9. 175-179 – If CL was involved in analysis, would her OPD experience not also be relevant here?

CL was not involved in the initial development of the themes but supported the broader conception of the study, access to services, staff and participants and helped review the final iterations of the work.

10. 186 – Is a mental health nurse really considered operational, rather than clinical, staff? Obviously, this would also change your results section, and how you denote participants.

The mental health nurse was conceptualised as operational, due to their tasks within the service aligning with that of the prison officers in relation to providing key work support to service users. We have added to line 215-217 to clarify this:

“The mental health nurses were part of the operational workforce, due to their role within PERS aligning with the operational delivery of the service, such as providing key work sessions”.

11. The overarching theme title for theme two feels geared towards staff actions/experience/attitudes, but this theme also seems to encapsulate how service-users see and approach staff differently in PERS services. This may work as a kind of virtuous circle (whereby the more trusting staff appear, the more service users meaningfully engage, thereby increasing how trusting staff appear....). I just wonder if the theme heading could more explicitly acknowledge this change in behaviour/attitude from SUs as well as staff.

Thank you for this thoughtful observation and suggestion. We have changed it to: “We’ve got some understanding of their journey”: increasing trust and meaningful engagement with service users” throughout the manuscript.

12. Just wonder, in the reflective statement, if there were any differences in the attitudes between service users still within PERS, and ex-service users, now in the community.

It was not apparent that current and ex (in the community) service users held any notably different views or attitudes. However, it is hypothesised that differing attitudes may be held by ex-service users who had been returned to closed conditions and this is therefore identified as a limitation within the discussion section.

13. 335 – ‘In others’......‘and in systems more widely!’

Thank you. On line 366 we have added this: The suspicion and reluctance to engage in PERS highlights potential negative perceptions of the service, which is likely linked to a lack of trust in others (33) and in systems more widely.

14. 336 - Indeed. I felt the themes were, to some extent, presented in chronological order, with seemingly initial mistrust towards PERS mitigated by development of trust, with this then influencing engagement in later stages of the ‘rehabilitation’ and ‘release’ process (e.g., parole). Again, can’t help but consider the idea of a ‘virtuous cycle’ here. Just a reflection!

Thank you for this reflection and engagement with the themes presented. We have utilised this reflection within the discussion section on line 434-438:

“The themes demonstrate initial mistrust towards PERS, mitigated by development of trust through support centred around their needs, with this trust then influencing engagement in later stages whereby PERS staff were able to use the therapeutic relationships they developed with service users to support them to progress through their sentence”.

15. Limitations – While I appreciate why additional demographic data were not collected, it would be helpful to acknowledge the absence of ethnicity information, particularly for SUs. Due to this omission, obviously the paper cannot comment on known demographic skews within OPD service user populations (typically over-representing white individuals) or consider potential differences in experiences across ethnic groups.

We have included on line 421-428: “It was also not possible to report demographic information, including ethnicity and therefore we were not able to comment on how experiences across may have differed across ethnic groups or the representativeness of the sample.”

16. 387-388 – Whilst I agree, I do think it would be important to caveat that this paper looked at quite a specific part of the prison estate.

Thank you. We agree and have included on line 435-437: “Despite this study exploring low security open prisons, it is promising that the provision of individualised support, addressing needs on a person-by-person basis was received positively by service users and staff; while our findings cannot necessarily be generalised beyond this setting, findings may inform approaches across the prison estate.”

Reviewer #2: Thank you for the opportunity to review this paper, and my congratulations to the authors on this novel work to explore the experiences of staff and service users of supports for people in open prisons with personality disorders. Below I have included some thoughts for your consideration, I hope they are helpful.

1. Acknowledging the exception for the name of programs (such as the OPD pathway), I strongly recommend the authors replace the word “offender(s)” throughout the paper and instead use a person-centred alternative, consistent with practices for reducing stigma, prejudice and harm in research and healthcare. (for example, in the abstract, first line of the introduction, page 4 line 78…) The papers below may be helpful resources.

Tran NT, Baggio S, Dawson A, et al. Words matter: a call for humanizing and respectful language to describe people who experience incarceration. BMC International Health and Human Rights. 2018.

Bedell PS, Spaulding AC. The Names Have Been Changed to Protect the . . . Humanity: Person-First Language in Correctional Health Epidemiology. American Journal of Epidemiology. 2018.

Thank you for your positive and helpful comments. We now refer to ‘people in prison’ or ‘individuals’ or removing ‘offender’ where it was not needed (it is clear the people being referred to are in prison). Please see tracked changes throughout the manuscript.

Introduction

2. P5L96 This sentence uses “high-risk” twice, it’s not clear to me what the first use to label people means. If the authors could please revise to clarify who the program is meant to serve (“high risk” individuals is also used on P4L87, if the authors could please clarify, high risk of/for what?).

We have rewritten the sentence for improved clarity of what the risk is of/for on P5 line 115-119:

“Open prisons are a particularly challenging environment for individuals with a likely diagnosis of personality disorder and therefore open prison OPD services known as Pathways Enhanced Resettlement Services (PERS) have been operational since 2019, focusing on individuals with a high risk of “failing” in the short-term, either through reoffending following release or being returned to closed conditions”.

We have further clarified what the high risk is of/what for on P5L105-106: “The pathway was set up in 2012 to support individuals with a high risk of recidivism, assessed as meeting criteria for personality disorder”.

Methods

3. More details on participants selection and the process of conducting interviews and informed consent are needed (Please see COREQ items 9-23). A few key examples of needed details:

- How were staff members selected and approached to participate? How many were invited and how many declined to participate?

- How were potential participants selected and invited to participate? How many people declined to participate? How was informed consent ensured (was the consent form reviewed verbally?). How was confidentiality protected during the interviews?

- Were people compensated for their participation?

Thank you for noticing these omissions, we have reviewed the COREQ along with the prompts you have provided here and added in details throughout the methods section. Specifically:

How were staff members selected and approached to participate? How many were invited and how many declined to participate?

On line 183- 188 we added:

“we recruited ten of twenty staff (two staff members from each service were approached via email to take part in the study), purposively, for diversity of staff roles, levels of experience and clinical and operational staff. All staff that were approached to take part in the study agreed to participate…”

How were potential participants selected and invited to participate? How many people declined to participate?

On line 190- 196 we added:

“Potential participants were initially approached by PERS staff, directly or via offender managers of those returned to closed conditions between 7th May 2022 and 1st September 2022. Staff then arranged interviews for current service users and passed details of ex-service users living in the community willing to participate to the researcher. It was not possible to approach anyone in closed conditions prisons during the study period due to COVID restrictions and therefore none took part in the study. All participants who were approached by PERS staff took part in the study.”

How was informed consent ensured (was the consent form reviewed verbally? How was confidentiality protected during the interviews?

On line 187 – 188 we added:

“…provided informed consent by completing the consent form and emailing it to the lead author before the interview began.”

On line 196-201 we added:

“Informed consent was obtained in person prior to the interview for current service users who were interviewed in person allowing opportunity to clarify anything before beginning. Individuals in the community completed the consent form and returned it to the lead researcher via email before the interview commenced and at the start of the interview this was verbally re-confirmed.”

- Were people compensated for their participation?

On line 203 we have added: “Neither staff nor service users received any compensation for their participation.”

4. (L154) The data analysis section is written in the plural first person, but there is no stage that describes any author besides GM being engaged in the work. Multiple coders are not required by RTA but suggest revising to be more explicit that there was no other input or review

We have provided further clarification of who undertook each stage of the analysis on P8L212-213: GM carried out phase 1-3 of the RTA. The initial themes were developed, reviewed and refined alongside MF and HJ.

5. (L171) I think it would be helpful for authors to include why member checking was conducted only with staff and not program participants.

Thank you we have included the following sentence on P9L215-6: It was not possible to re-contact service users to check the themes.

6. The section on researcher positionality requires more details to be consistent with COREQ guidelines:

- Specifically more details are required about what it means that co-authors “previously worked’ in OPD pathway services (as staff or care providers?)

-

# Editor report: Decision Letter 1

**DOI:** 10.1371/journal.pone.0350292.r003

**Authors:** Steph Scott

13 May 2026

Exploring staff and service user experiences of personality disorder services in open prisons: A qualitative study of Pathways Enhanced Resettlement Support

PONE-D-25-20906R1

Dear Dr. Mathlin,

We’re pleased to inform you that your manuscript has been judged scientifically suitable for publication and will be formally accepted for publication once it meets all outstanding technical requirements.

Within one week, you’ll receive an e-mail detailing the required amendments. When these have been addressed, you’ll receive a formal acceptance letter and your manuscript will be scheduled for publication.

An invoice will be generated when your article is formally accepted. Please note, if your institution has a publishing partnership with PLOS and your article meets the relevant criteria, all or part of your publication costs will be covered. Please make sure your user information is up-to-date by logging into Editorial Manager at Editorial Manager® and clicking the ‘Update My Information' link at the top of the page. For questions related to billing, please contact billing support.

If your institution or institutions have a press office, please notify them about your upcoming paper to help maximize its impact. If they’ll be preparing press materials, please inform our press team as soon as possible -- no later than 48 hours after receiving the formal acceptance. Your manuscript will remain under strict press embargo until 2 pm Eastern Time on the date of publication. For more information, please contact onepress@plos.org.

Kind regards,

Steph Scott

Academic Editor

PLOS One

Additional Editor Comments (optional):

Reviewers' comments:

# Editor report: Acceptance letter

**DOI:** 10.1371/journal.pone.0350292.r004

**Authors:** Steph Scott

PONE-D-25-20906R1

PLOS One

Dear Dr. Mathlin,

I'm pleased to inform you that your manuscript has been deemed suitable for publication in PLOS One. Congratulations! Your manuscript is now being handed over to our production team.

At this stage, our production department will prepare your paper for publication. This includes ensuring the following:

* All references, tables, and figures are properly cited

* All relevant supporting information is included in the manuscript submission,

* There are no issues that prevent the paper from being properly typeset

You will receive further instructions from the production team, including instructions on how to review your proof when it is ready. Please keep in mind that we are working through a large volume of accepted articles, so please give us a few days to review your paper and let you know the next and final steps.

Lastly, if your institution or institutions have a press office, please let them know about your upcoming paper now to help maximize its impact. If they'll be preparing press materials, please inform our press team within the next 48 hours. Your manuscript will remain under strict press embargo until 2 pm Eastern Time on the date of publication. For more information, please contact onepress@plos.org.

You will receive an invoice from PLOS for your publication fee after your manuscript has reached the completed accept phase. If you receive an email requesting payment before acceptance or for any other service, this may be a phishing scheme. Learn how to identify phishing emails and protect your accounts at https://explore.plos.org/phishing.

If we can help with anything else, please email us at customercare@plos.org.

Thank you for submitting your work to PLOS ONE and supporting open access.

Kind regards,

PLOS ONE Editorial Office Staff

on behalf of

Dr. Steph Scott

Academic Editor

PLOS One
